# Felty综合征伴惰性γδT细胞克隆1例报告并文献复习

**DOI:** 10.3760/cma.j.cn121090-20250627-00302

**Published:** 2026-02

**Authors:** 聪 王, 明伟 傅, 刚 安, 文阳 黄, 莉洁 杨

**Affiliations:** 1 中国医学科学院血液病医院（中国医学科学院血液学研究所），血液与健康全国重点实验室，国家血液系统疾病临床医学研究中心，细胞生态海河实验室，天津 300020 State Key Laboratory of Experimental Hematology, National Clinical Research Center for Blood Diseases, Haihe Laboratory of Cell Ecosystem, Institute of Hematology & Blood Diseases Hospital, Chinese Academy of Medical Sciences & Peking Union Medical College, Tianjin 300020, China; 2 西安国际医学中心医院血液科，西安 710000 Department of Hematology, Xi'an International Medical Center Hospital, Xi'an 710000, China

## Abstract

惰性γδT细胞克隆在Felty综合征（FS）中的动态演变及其与脾进行性肿大之间的关系尚未明确。本文通过十年的随访，报道一例FS患者病例资料，动态监测γδT细胞克隆负荷、脾大小及血细胞水平，发现患者十年间γδT细胞克隆负荷波动幅度小，而脾呈进行性增大，血细胞进行性减少，PET-CT新发现的鼻咽部高代谢灶（SUVmax 10.7）经病理检验证实为反应性浆细胞增生。本研究提示FS中可能存在γδT细胞克隆惰性增殖现象，其脾肿大进展机制或独立于淋巴细胞克隆。

## Abstract (en)

The dynamic evolution of indolent γδT-cell clones in Felty syndrome（FS）and their relationship with progressive splenomegaly remain unclear. This paper reports a case of an FS patient followed for ten years. Serial assessments of γδT-cell clone burden, spleen size, and blood cell levels were performed. The results revealed that the γδT-cell clone burden showed minimal fluctuation over the decade, while the spleen demonstrated progressive enlargement, and blood cells progressively decreased. A newly identified nasopharyngeal hypermetabolic lesion（SUVmax 10.7）on PET-CT was pathologically confirmed as reactive plasmacytosis. This study suggests that indolent proliferation of γδT-cell clones may exist in FS, and the mechanism underlying the progression of splenomegaly might be independent of the lymphocyte clone.

γδT细胞克隆增殖在世界卫生组织（WHO）发布的造血与淋巴组织肿瘤分类第5版中属于T细胞大颗粒淋巴细胞白血病（T-LGLL）范畴内的罕见亚型[1]，而自身免疫性疾病相关的γδT细胞惰性亚型未见系统描述。自身免疫微环境影响γδT细胞功能，但γδT细胞亚型参与自身免疫微环境构建过程中所表现的生物学特性尚未明确[2]。Felty综合征（FS）患者中γδT细胞克隆动态变化的数据较少，脾进行性增大是否与T细胞克隆相关仍未知[3]。本研究通过对1例FS患者的10年随访，探讨γδT细胞克隆的动态演变特点及其与脾进行性肿大之间的关联。

## 病例资料

患者，女，47岁，2025年5月14日因“发现白细胞减少10年，发热1个月”就诊于中国医学科学院血液病医院（中国医学科学院血液学研究所）。患者既往有41年的类风湿关节炎（RA）病史，1984年出现多关节对称性游走性疼痛，以双侧腕关节、掌指关节为主，实验室检查类风湿因子阳性，根据当时标准诊断为“青少年类风湿关节炎”，经药物治疗（具体不详）2年后好转，目前遗留双手远端指尖关节畸形。2015年无明显诱因反复出现咽痛、流涕伴发热（最高体温38.6 °C）、眼干、口干、反复口腔溃疡，就诊于当地医院，血常规：WBC 1.30×10^9^/L、ANC 0.42×10^9^/L、HGB 113 g/L、PLT 152×10^9^/L；骨髓细胞形态：粒系增生活跃，早幼粒细胞比例增高，浆细胞比例增高；染色体核型分析：未见异常；流式细胞术首次检出CD3^+^CD5^dim^TCRγδ^+^T淋巴细胞，约占CD3^+^T细胞的39.20％。当地医院考虑风湿免疫相关，诊断为干燥综合征（SS），给予沙利度胺（100 mg，每日1次）、甲泼尼龙（40 mg，每日1次）治疗，患者自诉监测血常规指标略好转（具体未见），2个多月后甲泼尼龙逐渐减量至每日8 mg，此后从6 mg减至5 mg时出现粒细胞减少加重，患者自行口服中药并将甲泼尼龙减量至4 mg维持治疗3年。

2018年1月3日患者于当地医院常规复查，血常规：WBC 0.95×10^9^/L、ANC 0.08×10^9^/L、RBC 3.85×10^12^/L、PLT 105×10^9^/L；红细胞沉降率29 mm/h；C反应蛋白12.50 mg/L；免疫球蛋白（Ig）G 23.56 g/L；抗核抗体胞浆型滴度1∶100；抗环瓜氨酸肽抗体>200 RU/ml；类风湿因子<10.40 IU/ml；抗链球菌溶血素O 40.30 IU/ml；超声：脾大（长径为16.5 cm）、门静脉高压伴肝脏形态改变及颈部淋巴结增大，但骨髓流式细胞术、甲状腺功能及感染筛查均未见异常。诊断：肝脾γδT细胞淋巴瘤待排；SS；RA；脾功能亢进。给予重组人粒细胞刺激因子（150 µg，每日1次）、硫酸羟氯喹（0.2 g，每日2次）、环孢素（50 mg，每日2次）、甲泼尼龙（40 mg，每日1次）等对症治疗，效果欠佳。当地医院建议患者到上级医院就诊行脾切除治疗及脾病理活检明确诊断。2018年1月10日患者就诊于北京某医院，血常规：WBC 2.73×10^9^/L、ANC 1.49×10^9^/L、HGB 113 g/L、PLT 130×10^9^/L；抗环瓜氨酸肽抗体>3 200 RU/ml；抗核周因子阳性；抗角蛋白抗体阳性；抗β_2_糖蛋白1抗体70 RU/ml；EB病毒DNA定量、红细胞沉降率、免疫固定电泳均未见异常；PET-CT示中央及外周骨髓代谢普遍增高［标准摄取值（SUV）2.6～3.8］，脾大且代谢增高（脾长径16.5 cm，SUV为2.1～2.9）；骨髓穿刺：异型淋巴细胞比例为3.50％；骨髓流式细胞术未检出异常表型细胞。临床医师建议随诊，3个多月后甲泼尼龙片逐渐减至4 mg，患者口服甲泼尼龙片疗效欠佳，2020年自行停用激素，此后患者因无不适症状未再复查，未再接受系统性免疫抑制治疗。

2025年5月患者因病情变化来我院就诊。血常规：WBC 0.37×10^9^/L、ANC 0.15×10^9^/L、HGB 61 g/L、平均红细胞体积87.60 fl、PLT 98×10^9^/L。外周血涂片：白细胞减少，粒细胞比例下降，细胞形态大致正常；成熟红细胞大小不一，计数100个白细胞未见有核红细胞；淋巴细胞比例增高，可见约11％不典型淋巴细胞，形态同骨髓；单核细胞比例增高，为成熟单核细胞；血小板单个、散在分布，易见。外周血流式细胞术：成熟淋巴细胞占有核细胞的36.75％，CD3^+^ T细胞占淋巴细胞的89.42％，CD3^+^CD4^+^/CD3^+^CD8^+^细胞比值为2.80，CD3^-^CD16^+^/CD56^+^NK细胞占淋巴细胞的3.60％，CD3^+^CD56^+^细胞占淋巴细胞的2.81％，CD3^+^CD57^+^T细胞占淋巴细胞的3.42％，CD19^+^成熟B细胞占淋巴细胞的5.99％。Kappa/Lambda＝1.72；淋巴细胞各亚群比例及表型未见明显异常。骨髓检查：骨髓形态：可见约8％不典型淋巴细胞，该类细胞胞体中等或偏大，核近圆形或不规则，染色质偏疏松，偶见核仁，胞质量少，边缘不整，部分细胞可见颗粒。活检：T淋巴细胞增殖性疾病。流式细胞术：成熟淋巴细胞占有核细胞的19.96％，CD3^+^T细胞占淋巴细胞的91.90％，异常细胞群占有核细胞的5.73％，强表达CD3，表达TCRγδ、CD7、CD45RA，弱表达Perforin，不表达CD5、CD4、CD8、TCRγδ1/γδ2、CD57、GranzymeB、CD56、CD16、CD45RO、CD30、CD26；结论：淋巴细胞各亚群比例如上，TCRγδ^+^T细胞约占T细胞的31.78％，比例增高，FSC及SSC偏大，不表达γδ1及γδ2。B细胞、NK细胞表型未见明显异常。染色体核型分析：未见克隆性异常，无i（7q）/+8。FISH检测：BCL2、BCL6、MYC、IGH均阴性。TCRδ重排阳性。TCRγ重排阴性。二代测序：Ⅰ级突变TET2 p.C1221Y 7.71％；Ⅲ级突变TYK2 p.R700W 48.36％。

PET-CT：脾大，大小为27.4 cm×15.3 cm×0.7 cm，SUVmax为3.1；骨骼弥漫代谢增高，SUVmax为5.1；考虑淋巴造血系统恶性肿瘤可能（Deauville评分：5分）；左鼻腔黏膜增厚，SUVmax约为7.6；鼻咽及口咽壁增厚，SUVmax约为10.7。鼻部病灶的流式细胞术检测显示TCRγδ^+^T细胞占T细胞的2.10％；病理：淋巴组织增生伴浆细胞明显增多，免疫组化示κ阳性细胞多于λ阳性细胞；免疫组化：CD3、CD5、CD20少量阳性表达，CD19较多阳性表达，CD38阳性表达，浆细胞κ部分阳性、λ少部分阳性表达，Ki-67阳性率约10％，cyclinD1阴性、CD56少量阳性表达。

## 讨论及文献复习

本文通过十年随访，在FS患者中观察到γδT细胞克隆惰性增殖，脾进行性增大和克隆负荷变化分离现象，对FS的异质性有新的认识。本例患者骨髓中存在高比例且惰性增殖的γδT细胞克隆（31.78％），而外周血比例仅为4.11％，鼻腔组织也仅检出2.10％，提示本例γδT细胞克隆主要富集并局限于骨髓微环境。这与血液系统肿瘤领域部分现象相似，如急性髓系白血病患者骨髓中γδT细胞相关免疫调节受体（如TIGIT、TIM-3）表达水平明显升高[4]，淋巴瘤和多发性骨髓瘤患者骨髓中γδT-1细胞比例显著高于外周血[5]，且免疫调节受体（如TIGIT、PD-1、TIM-3或CD39）可能与克隆的惰性状态密切相关[6]。本研究在FS中观察到骨髓γδT克隆的惰性高表达，这提示骨髓可能是驱动异常免疫应答或维持异常克隆的关键场所，未来可通过检测细胞亚型（Vδ1/Vδ2）及TIGIT等受体表达进一步验证。

在免疫相关背景下，患者由单纯粒细胞减少逐渐进展为全血细胞减少，脾进行性增大，而γδT细胞克隆负荷十年间幅度波动小，提示驱动脾进展和血细胞减少的核心机制可能独立于淋巴细胞克隆的短期波动，而主要由自身免疫微环境驱动的脾结构性重构所介导。其潜在机制包括：①自身免疫性脾功能亢进：FS中脾巨噬细胞过度活化可吞噬血细胞并释放炎症因子来抑制中性粒细胞的循环[7]。本例采用激素治疗后，克隆短暂消失而脾进行性增大，提示巨噬细胞活化可能不依赖于T细胞的自我维持状态。②纤维化驱动：长期炎症微环境可以使脾基质重构，即使脾的SUVmax较低（如本例脾SUVmax仅3.1），脾仍可能表现出进展性病变，这提示脾进展可能受到局部微环境的调控如纤维化和基质细胞的重塑[8]。③细胞因子风暴：本例患者携带TYK2 p.R700W突变，其可能通过JAK-STAT通路，促进炎症反应，直接刺激脾基质细胞增殖[9]。

本例患者行PET-CT检查发现鼻咽SUVmax为10.7，初诊疑似淋巴瘤浸润，后经病理检查确诊为多克隆浆细胞反应性增生，鼻咽部组织γδT细胞占2.10％，这提示自身免疫性疾病患者的高代谢灶并非均是肿瘤性疾病，更多的是反映机体对局部病灶的炎性反应，其可能是血清中抗体水平升高导致的。PET-CT SUVmax无法完全反映病变的生物学本质，有研究报道淋巴瘤与成人Still病PET-CT SUVmax并无显著差异[10]。提示在某些病理状态下，病灶的代谢活性可能受到抑制，使得PET-CT成像的敏感性降低。因此，自身免疫性相关淋巴细胞增殖性疾病中高代谢灶通常是炎症反应而非恶性转化，其诊断需组织病理学明确，同时可通过流式细胞术分析免疫细胞的亚群分布和功能状态，从而判断是否存在免疫异常或恶性转化的迹象[11]。

本例需与T-LGLL鉴别，3％～28％的T-LGLL患者伴有RA，两者在临床和分子学特征上存在相似性，以及在免疫学和遗传学上存在联系[12]，但在诊断上仍存在差异。主要鉴别点为细胞起源与免疫表型不同：T-LGLL通常表现为持续性大颗粒淋巴细胞增多，绝大多数T-LGLL起源于αβT细胞（CD3^+^CD4^-^CD8^+^CD56^-^CD57^+^），少数T-LGLL起源于γδT细胞，γδT-LGLL多由双阴性（CD4^-^/CD8^-^）γδT细胞选择性增殖所致，表现为CD3^+^CD4^+/-^CD8^-^CD57^+^等不同表型[13]；而本例患者外周血涂片未见典型大颗粒淋巴细胞，外周血流式细胞术未检测到CD8^+^CD57^+^的大颗粒淋巴细胞群体，骨髓免疫表型为γδT细胞（CD3^+^CD4^-^CD8^-^CD56^-^CD57^-^）异常增殖。综合其惰性病程与免疫表型，本例支持FS相关的特殊类型惰性γδT细胞克隆性增殖诊断。

本例患者的病历资料显示了FS的特殊病程即惰性克隆和脾进行性肿大，其提示临床管理需注意：随访观察克隆和脾的变化，脾进展速度比克隆负荷更能反映疾病严重程度，正确判断肿瘤和良性炎症，需病理+流式细胞术+突变谱等联合检测以减少漏诊误诊。另外，治疗更应该重视对脾微环境的调节，采用免疫调控或抗纤维化、细胞因子通路如JAK抑制剂治疗可能比单纯靶向淋巴细胞更有效。本例患者经激素治疗后克隆短暂消失，提示免疫调控治疗可能有效，因血细胞恢复欠佳自行停用激素5年，考虑可能存在血细胞恢复的滞后性。结果提示在未规范治疗干预情况下，病程长达10年，更加验证了长期随访的关键性。本例未获取脾病理标本，无法直接观察γδT细胞在脾内空间分布和与周围微环境相互作用；自身免疫抗原驱动γδT细胞克隆增殖的具体分子机制尚未明确，个案报道难以确证现象普适性，未来需扩大样本进一步验证。
